# O052: Use of surgical-site infection rates to rank hospital performance across several types of surgery

**DOI:** 10.1186/2047-2994-2-S1-O52

**Published:** 2013-06-20

**Authors:** AM van Dishoeck, MB Koek, EW Steyerberg, BH van Benthem, MC Vos, HF Lingsma

**Affiliations:** 1 Centre of Medical Decision Making, Department of Public Health, Erasmus MC, Rotterdam, the Netherlands; 2 Epidemiology and Surveillance, RIVM, Bilthoven, the Netherlands; 3 Department of Medical Microbiology and Infectious Diseases, Erasmus MC, Rotterdam, the Netherlands

## Introduction

Comparing and ranking hospitals based on health outcomes is becoming increasingly popular. Outcome measures such as SSI rates are being used more and more to compare hospitals’ performance using league tables and rank orders. Observed differences between hospitals may however be partly explained by random variation and by differences in case mix, causing concerns aboutthe validity of such hospital comparisons.

## Objectives

To explore whether surgical-site infection (SSI) rates are suitable for comparing hospitals, taking into account case-mix differences and random variation.

## Methods

Data from the national surveillance network in the Netherlands, on the eight most frequently registered types of surgery for the year 2009, were used to calculate SSI rates. The variation in SSI rates between hospitals was estimated with multivariable fixed- and random-effects logistic regression models to account for random variation and case mix. ‘Rankability’ (as the reliability of ranking) of the SSI rates was calculated by relating within-hospital variation to between-hospital variation.

## Results

Thirty-four hospitals reported on 13 629 patients, with overall SSI rates per surgical procedure varying between 0 and 15.1 per cent. Statistically significant differences in SSI rate between hospitals were found for colon resection, caesarean section and for all operations combined. Rankability was 80 per cent for colon resection but 0 per cent for caesarean section. Rankability was 8 per cent in all operations combined, as the differences in SSI rates were explained mainly by case mix.

## Conclusion

When comparing SSI rates in all operations, differences between hospitals were explained by case mix. For individual types of surgery, case mix varied less between hospitals, and differences were explained largely by random variation. Although SSI rates may be used for monitoring quality improvement within hospitals, they should not be used for ranking hospitals.

## Disclosure of interest

None declared.

